# Genome-wide identification, expression and function analysis of the *MTP* gene family in tulip (*Tulipa gesneriana*)

**DOI:** 10.3389/fpls.2024.1346255

**Published:** 2024-02-19

**Authors:** Jiaojiao Lu, Guimei Xing, Yanqiu Zhang, Huihua Zhang, Tianyu Wu, Zengzhi Tian, Lianwei Qu

**Affiliations:** ^1^ Institute of Floriculture, Liaoning Academy of Agriculture Sciences, Shenyang, Liaoning, China; ^2^ Liaoning Provincial Key Laboratory of Floriculture, Shenyang, Liaoning, China

**Keywords:** *Tulipa gesneriana*, heavy metal accumulation, metal tolerance proteins (MTPs), phylogenetic analysis, gene expression regulation

## Abstract

Currently, soil heavy metal contamination is a severe issue, particularly with Cd pollution. The metal tolerance protein (MTP) proteins, as plant divalent cation transporters, play a crucial role in the transport and tolerance of heavy metals in plants. This study conducted comprehensive identification and characterization of the *MTP* gene family in the tulip. A total of 11 *TgMTP* genes were identified and phylogenetically classified into three subfamilies. Conserved motif and gene structure analyses unveiled commonalities and variations among subfamily members. Expression profiling demonstrated several *TgMTP*s were markedly upregulated under Cd exposure, including the *TgMTP7.1*. Heterologous expression in yeast validated that *TgMTP7.1* could ameliorate Cd sensitivity and enhance its tolerance. These results provide primary insights into the *MTP* gene family in tulip. Phylogenetic relationships and functional analyses establish a framework for elucidating the transporters and molecular mechanisms governing Cd accumulation and distribution in tulip. Key *TgMTP*s identified, exemplified by *TgMTP7.1*, may illuminate molecular breeding efforts aimed at developing Cd-tolerant cultivars for the remediation of soil Cd contamination.

## 1 Introduction

Tulip (*Tulipa gesneriana*) is one of the most important ornamental geophytes, cultivated worldwide for its spectacularly colored flowers. As the third largest cut flower after rose and chrysanthemum, the global production of tulip has significant market value (Byczyńska et al., 2019). However, with industrial development, heavy metal pollution has become an increasing concern, ornamental plants grown along roadsides are often subjected to heavy metal stress, tulip also belongs to ornamental plants (Dodangeh et al., 2018). There are significant differences in the accumulation of cadmium and lead between different genotypes of plants or different organs in tulipa (Alexander et al., 2006; Osmani et al., 2018). Excessive accumulation of these non-essential metals could lead to inhibited plant growth, reduced bulb yield and quality through oxidative damage, genotoxicity and interference with nutrient homeostasis (Djebali et al., 2005; Liu et al., 2008).

To cope with metal stress, plants have evolved a complex metal homeostatic network consisting of various membrane transporters and chelators that tightly regulate the uptake, efflux, sequestration, trafficking and detoxification of metal ions (Hall and Williams, 2003). The cation diffusion facilitator (CDF) family, also known as metal tolerance proteins (MTPs) in plants, plays pivotal roles in this network by mediating efflux or sequestration of diverse metal ions across biomembranes (Gustin et al., 2011). There are three subfamilies of MTPs in plants, contain Mn-CDF, Fe/Zn-CDF, and Zn-CDF, and they were classified into seven groups in *Arabidopsis* (Montanini et al., 2007; Gustin et al., 2011). Plant MTPs function as H^+^/metal-ion antiporters driven by proton gradients. They contribute to metal tolerance by sequestration of non-essential toxic metals like Cd^2+^ and Ni^2+^ into vacuoles. Their transport of essential metals like Zn^2+^, Mn^2+^, Fe^2+^ and Co^2+^ also facilitates normal growth and development (Paulsen and Saier, 1997; Haney et al., 2005). ShMTP1 conferred Mn^2+^ tolerance through internal sequestration (Delhaize et al., 2003). The *ZAT* gene of *Arabidopsis* confers increased Zn^2+^ tolerance when overexpressed in plants (Van der Zaal et al., 1999).

The *MTP* gene family has been investigated in several plant species, including *Arabidopsis thaliana* (Van der Zaal et al., 1999), rice (Ram et al., 2019), grape (Shirazi et al., 2019) and soybean (El-Sappah et al., 2023), common bean (Yilmaz et al., 2023) and tomato (El-Sappah et al., 2021). And many MTPs function have been identification, such as the heterologous expression of *CsMTP8.2* in *A. thaliana* could decrease the accumulation of Mn in plants (Zhang et al., 2020), the heterologous expression *GmMTP8.1* restored growth of Mn-hypersensitive yeast mutant Δ*pmr1* (Li et al., 2021). Meanwhile, it is noteworthy that phylogenetic analyses have classified plant MTPs into three major subfamilies, but substrate specificities are not absolute, as some MTPs transport multiple metals (Montanini et al., 2007). *MTP* genes also show differential tissue-specific and metal-responsive expression patterns (Hassinen, 2009). Overexpression of particular *MTP*s has been found to increase tolerance to Zn, Co, Ni, Fe and Cd (Montanini et al., 2007; Chen et al., 2013; Menguer et al., 2013). In tobacco, under heavy metal toxicity, the *NtMTP* gene exhibited distinct responses in tobacco leaves and root systems. *NtMTP8.1*, *NtMTP8.4* and *NtMTP11.1* displayed Mn transport capability in yeast cells (Liu et al., 2019). In rice, *OsMTP11* may play an important role in Mn and other heavy metal transport in rice (Zhang and Liu, 2017). In *Eucalyptus grandis*, The up-regulation of *EgMTP6*, *EgMTP5* and *EgMTP11.1* upon excessive exposure to Cd^2+^ and Cu^2+^ may account for the metal translocation from root to leaf (Shirazi et al., 2023). In *Populus trichocarpa*, *PtrMTP8.1*, *PtrMTP9* and *PtrMTP10.4* displayed Mn transport capability in yeast cells, while *PtrMTP6* was able to transport Co, Fe and Mn (Gao et al., 2020).

Given the importance of MTP transporters in plant metal homeostasis, characterizing the *MTP* gene family in tulip will provide critical insights into the mechanistic basis of heavy metal accumulation and distribution in this species. Genome-wide identification of MTPs in tulip may also present valuable targets for developing cultivars tolerant to metal stresses. Moreover, as an ornamental plant, comprehensive identification of MTPs in tulip will facilitate its application in restoring heavy metal polluted soils, like other ornamental plants (Dodangeh et al., 2018). However, genome-wide analysis and functional characterization of MTPs has not yet been performed in tulip.

In this study, we identified and comprehensively analyzed the *MTP* gene family in tulip through Iso-seq mining and phylogenetic comparisons with *A. thaliana* and rice MTPs. Gene and protein structures were determined, along with conserved motifs and phylogenetic relationships. Expression profiles of *TgMTP*s were examined using RNA-seq data. Several MTPs showing metal responsiveness were selected for functional analyses via heterologous expression in yeast. Yeast complementation assays tested Cd transport capabilities. This multifaceted investigation of the *MTP* gene family provides the first insights into the molecular basis of heavy metal accumulation and tolerance mechanisms in tulip. This study is expected to enhance the understanding of TgMTPs’ functions and establish a solid foundation for scrutinizing the roles and mechanisms of TgMTP proteins.

## 2 Methods and materials

### 2.1 Identification of *TgMTP* gene family members in tulip

Based on the full-length transcriptome assembly results, the CDS sequence, protein sequence, and annotation file of the genome were obtained (bioproject_accession: PRJNA1028662). The Pfam numbers PF16916 and PF01545 of MTP were obtained from the InterPro (https://www.ebi.ac.uk/interpro/ database to download its Hidden Markov Model (HMM) file. The HMM file of MTP was used as input, and hmmsearch (v3.3.2) software (Johnson et al., 2010) was used to search the tulip protein sequence file with *E*-value < 0.05.

Secondly, the sequence files of MTP proteins in rice and *A. thaliana* were downloaded from Phytozome (https://phytozome-next.jgi.doe.gov/) database as submission files. Blastp (v2.13.0) (Kelley et al., 2004) was used to search for potential MTP proteins in tulip protein files, and the results of hmmsearch and blastp were combined to obtain candidate gene ID. The protein sequences of candidate genes were extracted and submitted to Pfam, CDD (https://www.ncbi.nlm.nih.gov/Structure/cdd/cdd.shtml) and SMART (http://smart.embl-heidelberg.de) database for domain confirmation to obtain the determined TgMTP protein.

### 2.2 Analysis of TgMTPs’ physical and chemical properties

By comparing with *A. thaliana* and rice MTP family members and naming *TgMTP* family members, the physicochemical properties of TgMTP family members were analyzed by Expasy (https://web.expasy.org/protparam/) software. Cell-PLoc-2 (http://www.csbio.sjtu.edu.cn/bioinf/Cell-PLoc-2/) software was used to predict the subcellular localization of TgMTP.

### 2.3 Conserved motifs and gene structure analysis of TgMTP protein

All MTP protein sequences of tulips were used as input files, and the conserved motifs of *TgMTP* family were analyzed by MEME software (version: v5.0.5, http://meme-suite.org/) (Bailey et al., 2006). The parameter motif search number is 10, the minimum motif amino acid length is 6, and the maximum motif amino acid length is 100. The R package gggenomes (https://thackl.github.io/gggenomes/) was used to visually analyze the conserved motif and domain of TgMTP, and TBtools was used to visualize the CDS and UTR regions. The signal peptide of TgMTP protein was predicted by SignalP (v5.0) software (https://services.healthtech.dtu.dk/service.php?SignalP-5.0).

### 2.4 Ka/Ks analysis of *TgMTP* gene family members

Ka/Ks analysis, a prevalent and crucial tool in bioinformatics, plays a significant role in examining the evolutionary dynamics of nucleic acid molecules. Within the realm of genetics, the Ka/Ks ratio signifies the balance between the nonsynonymous substitution rate (Ka) and the synonymous substitution rate (Ks) in two protein-coding genes. This metric serves as an indicator of selective pressure acting on these genes, using KaKs _ Calculator (v2.0) (Wang et al., 2010) software for analysis.

### 2.5 Phylogenetic analysis of *TgMTP* gene family members

The MTP family protein sequences of *A. thaliana*, rice, tomato and soybean were downloaded from the Phytozome database, and the phylogenetic tree was drawn using MEGA (v10) software (Kumar et al., 2018). Clustal W (Thompson et al., 1994) was selected as the comparison method, and the phylogenetic tree was constructed by Neighbor-joining method. The model was p-distance, the missing data method was Partial deletion, the cutoff was 50%, and Bootstrap was set to 1000. We use Evolview3 (https://www.evolgenius.info/evolview/) to beautify the tree.

### 2.6 Protein structure analysis and GO annotation of TgMTP

The protein secondary structure of TgMTP was predicted by sopma (https://npsa-prabi.ibcp.fr/cgi-bin/npsa_automat.pl?page=/NPSA/npsa_sopma.html) website, and the protein tertiary structure of TgMTP was predicted by SwissModel (https://swissmodel.expasy.org/). The clusterProfiler (Yu et al., 2012) in R was used to analyze the GO (Gene Ontology) annotation of *TgMTP*.

### 2.7 Regulation network analysis of miRNA- *TgMTP* gene family members

The psRNATarget online tool (https://www.zhaolab.org/psRNATarget/) was use to analysis the miRNA target gene network of *TgMTP* gene family. Additionally, since miRNA research in tulip remains scarce, we selected the model plant *A. thaliana*, which has the most extensive miRNA databases, to analyze the miRNAs regulating *TgMTP* genes. Cytoscape (Smoot et al., 2011) was used to visualize the interaction networks.

### 2.8 qRT-PCR analysis

Tulip cultivar “Dow Jones” was exposed at 5°C for 4 weeks and then plant them in the soil (growing media 4.0, Shangdao Co., Ltd, Jinan, China). After treatment, untreated tulip bulbs were used as CK group (0 d), and tulip bulbs were washed and placed in the medium containing different concentrations of metal ions (for FeSO_4_: 1 mM; for MnSO_4_: 5 mM; for ZnSO_4_: 1 mM and for CdCl_2_: 5 mM) in Hoagland nutrient solution. They were then cultivated in a conventional water-fertilizer incubator in soil with a 25°C/21°C day-night temperature and 16 h day^-1^ photoperiod. Bulbs subjected to these conditions were sampled at 1, 3, 5, and 7 days and total RNA was extracted using the BioRun Plant Total RNA Extraction Kit (Biorun Co., Ltd, Wuhan, China). cDNA was synthesized, followed by qRT-PCR to measure relative expression. Relative gene expression was calculated using the 2^-ΔΔCt^ method. Graphpad prism 9 software (https://www.graphpad.com/features) was used for plotting and statistical analysis. Primers were designed at NCBI and are listed in **Supplementary Table 1**.

### 2.9 Heterologous expression of *TgMTP7.1* in yeast

The heterogeneic expression of *TgMTP7.1* in yeast BY4741 and *ycf1*Δ were used to investigate whether *TgMTP7.1* can improve the Cd tolerance of yeast. The ORF of *TgMTP7.1* was amplified from tulip cDNA and inserted into the yeast expression vector pYES2 under the control of a galactose-inducible promoter. The resulting pYES2-*TgMTP7.1* and empty vector pYES2 were transformed into yeast cells. Transgenic yeast strains were cultured in synthetic complete (SC) medium lacking uracil with 2% (w/v) galactose to induce gene expression. For Cd tolerance assays, the exponentially growing yeast cultures were normalized to an OD_600_ of 0.2, and serial dilutions (10^0^, 10^1^, 10^2^, 10^3^) were prepared (Liu et al., 2019) 2 μL of each dilution was spotted onto SC-Ura agar medium containing 5 mM CdCl_2_. Plates were incubated at 30°C for 3-5 days before comparing growth differences. To ascertain metal tolerance, the growth of yeast (as measured by the OD_600_ value) was quantified at various intervals, employing methodologies delineated by Peng (Peng et al., 2018).

## 3 Results

### 3.1 Identification of *TgMTP* gene family members

Based on homologous sequence alignment and conserved domain analysis, a total of 11 *TgMTP* gene family members were identified in this study, named as *TgMTP1*, *TgMTP2*, *TgMTP3*, *TgMTP4*, *TgMTP5*, *TgMTP6.1*, *TgMTP6.2*, *TgMTP6.3*, *TgMTP7.1*, *TgMTP7.2* and *TgMTP7.3* (**Table 1**). The amino acid lengths (a. a. length) of the 11 TgMTP proteins ranged from 256 aa (TgMTP6.1) to 484 aa (TgMTP7.3). The theoretical isoelectric points (pI) and molecular weights (MW) of TgMTP proteins were 5.23 (TgMTP3 and TgMTP4) - 8.33 (TgMTP5) and 27576.85 Da (TgMTP6.1) - 51803.91 Da (TgMTP7.3), respectively. The grand average of hydropathicity (GRAVY) values distributed from -0.001 (TgMTP7.3) to 0.291 (TgMTP5). Except for TgMTP7.3 with a GRAVY of -0.001, all other members had GRAVY values greater than 0, indicating that the TgMTP family proteins are mostly hydrophobic, with only one member TgMTP7.3 being hydrophilic.

**Table 1:** Basic information of *TgMTP* gene family members.

Gene ID	Gene name	a. a. length	pI	MW	GRAVY
Unigene013504	*TgMTP1*	462	5.9	50113.87	0.056
Unigene009944	*TgMTP2*	420	5.81	45791.86	0.018
Unigene016125	*TgMTP3*	373	5.23	41910.48	0.092
Unigene018355	*TgMTP4*	373	5.23	41910.48	0.092
Unigene018015	*TgMTP5*	369	8.33	40500.92	0.291
Unigene032321	*TgMTP6.1*	256	7.41	27576.85	0.096
Unigene032835	*TgMTP6.2*	258	7.01	27899.26	0.118
Unigene027956	*TgMTP6.3*	258	7.37	27690.05	0.130
Unigene019728	*TgMTP7.1*	366	5.95	39492.10	0.213
Unigene012401	*TgMTP7.2*	437	6.76	47293.95	0.079
Unigene012254	*TgMTP7.3*	484	7.87	51803.91	-0.001

### 3.2 Phylogenetic relationships of TgMTP protein

**Figure 1:**
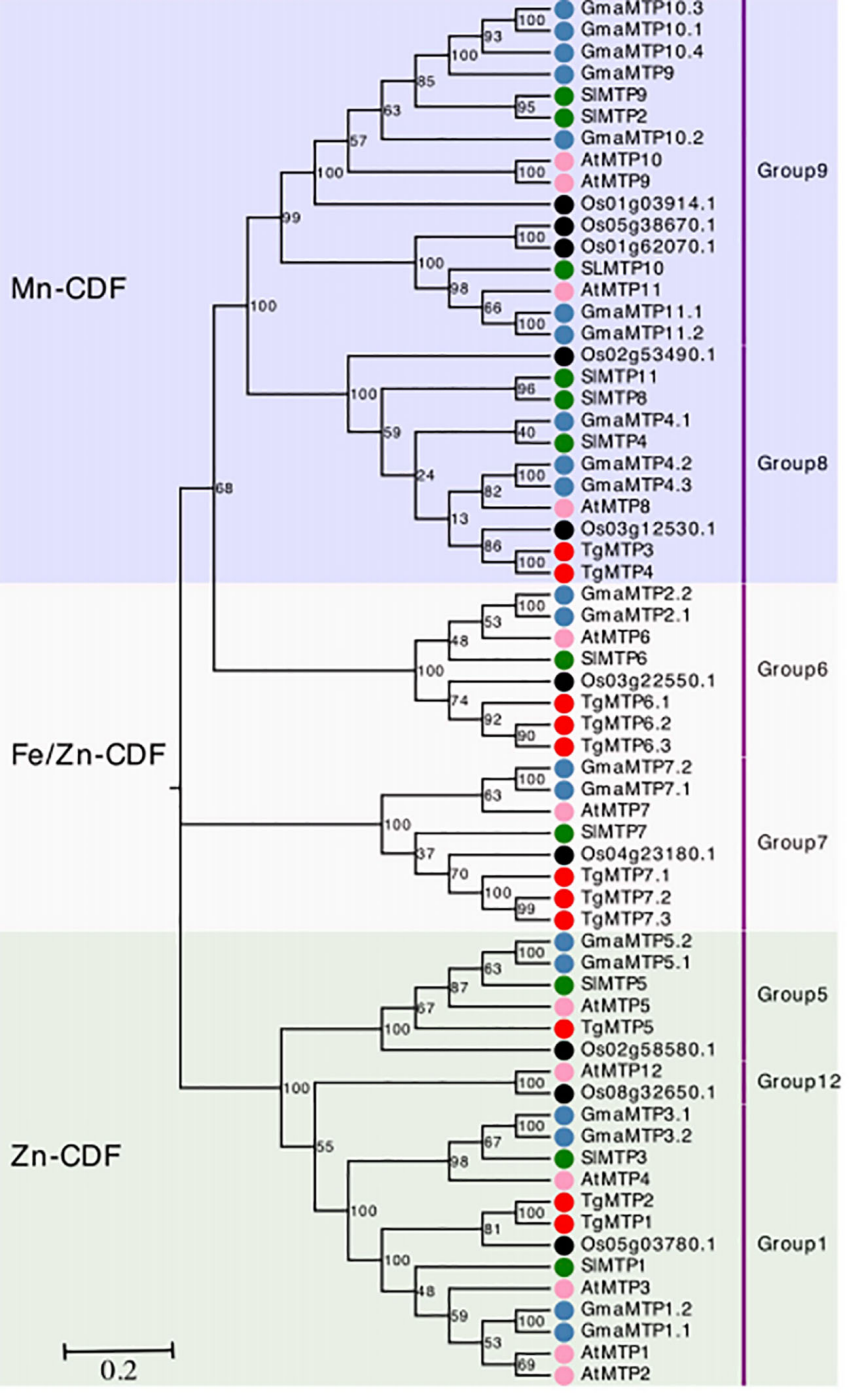
Phylogenetic relationships of TgMTPs with soybean (Gm), tomato (Sl), rice (Os) and *A. thaliana* (At) MTPs. The three clades were marked by different color. The MTP proteins are represented by solid circles with different colors denoting different plant species. Each group of MTP was annotated on right by text.

The phylogenetic relationships of TgMTPs with MTP members from soybean, tomato, rice and A. thaliana were displayed in the phylogenetic tree (**Figure 1**). The 63 MTP proteins were clustered into three major clades, designated as Mn-CDF, Fe/Zn-CDF and Zn-CDF subfamilies. The Mn-CDF clade contained TgMTP3 and TgMTP4. The Fe/Zn-CDF subfamily included 6 TgMTP members (TgMTP6.1-6.3, TgMTP7.1-7.3). TgMTP1, TgMTP2 and TgMTP5 belonged to the Zn-CDF group. Each clade also incorporated the homologous MTPs from the other plant species. The phylogenetic analysis revealed that the 11 TgMTPs were distributed among the 3 subfamilies together with respective orthologs. This suggests that the divergence of TgMTPs into different subfamilies preceded the speciation of tulip, soybean, tomato, rice and *A. thaliana*. The results provide insights into the evolutionary relationships and putative functional specificities of TgMTP transporters based on their classification into Mn-CDF, Fe/Zn-CDF and Zn-CDF subfamilies.

### 3.3 Structure and subcellular localization of MTP proteins

**Figure 2:**
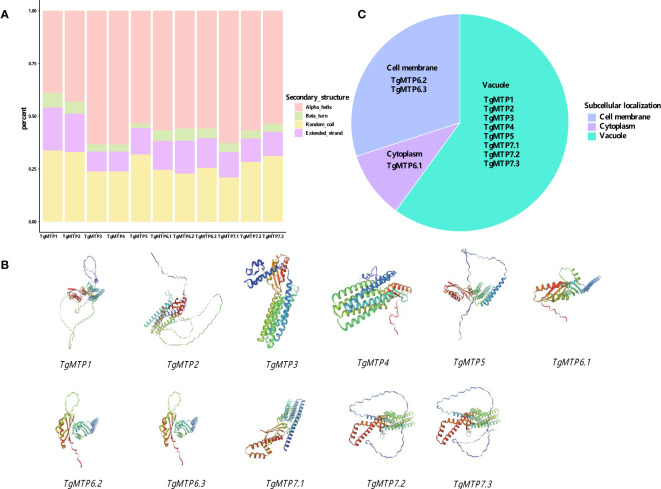
Structure and subcellular localization of MTP proteins. **(A)** The secondary structure composition of the 11 TgMTP proteins. **(B)** The predicted tertiary structures of the 11 TgMTP proteins. **(C)** The subcellular localizations of the 11 TgMTP proteins.

The predicted secondary structure composition of the 11 TgMTP proteins is summarized in **Figure 2A**. Four types of secondary structure elements were analyzed, including alpha helix, beta turn, random coil and extended strand. Among the TgMTPs, TgMTP3 and TgMTP4 contained the highest percentages of alpha helices at 63.27%, while TgMTP5 was enriched in extended strands at 12.47%. TgMTP7.1 exhibited the lowest content of random coils at 21.04%, whereas TgMTP7.3 showed the highest random coil percentage of 31.20%. The remaining TgMTPs had moderate compositions of the four secondary structures. In general, alpha helix appeared to be the predominant element in most TgMTPs, accounting for 38.96%-63.27% of their sequences. However, the proportions of alpha helices, beta turns, random coils and extended strands varied among different TgMTP members. These compositional divergences in secondary structures may contribute to functional specificities of the TgMTP transporters.

The predicted tertiary structures of the 11 TgMTP proteins are presented in **Figure 2B**. Homology modeling was performed using SWISS-MODEL with the best-match templates. The tertiary structures of TgMTPs displayed the typical architecture of MTP/CDF transporters, consisting of 6 transmembrane helices (TM1-6) and connecting loops.

The subcellular localizations of the 11 TgMTP proteins were predicted using Cell-PLoc-2 (**Figure 2C**). The results showed TgMTP1, TgMTP2, TgMTP3, TgMTP4, TgMTP5, TgMTP7.1, TgMTP7.2 and TgMTP7.3 were localized to the vacuolar membrane. TgMTP6.2 and TgMTP6.3 were predicted to target the plasma membrane. Whereas TgMTP6.1 was identified as a cytoplasmic protein.

### 3.3 Conserved motif, domains and CDS of TgMTP genes

**Figure 3:**
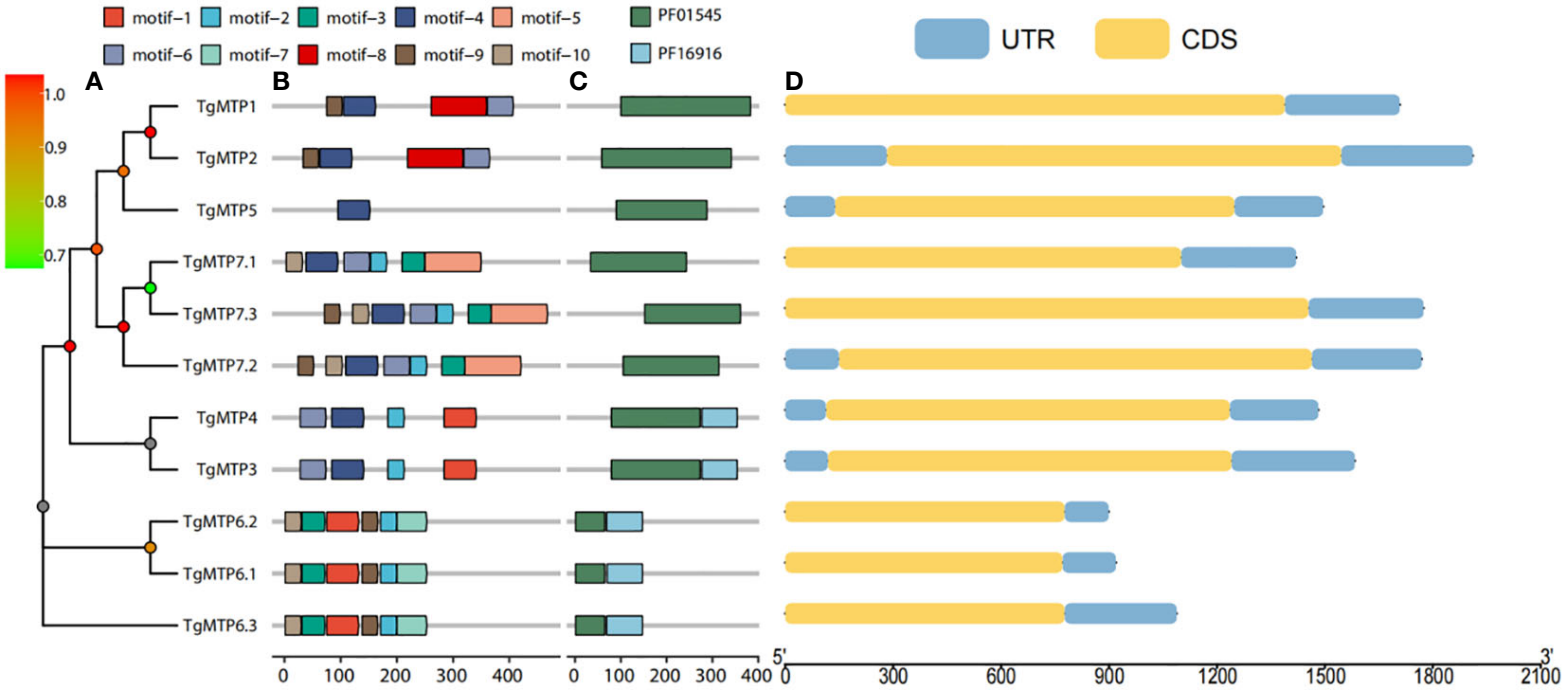
Phylogenetic tree **(A)**, conserved motifs **(B)** and conserved domains **(C)** of TgMTP proteins, and CDS **(D)** of *TgMTP* genes.

The phylogenetic relationship of *TgMTP* genes were shown in **Figure 3A**, the phylogenetic tree illustrates a high correspondence between evolutionary relationships and conserved motif as well as domain architectures. To better understand the diversity and similarity of *TgMTP* genes, conserved motifs in TgMTP proteins were analyzed using the MEME software (**Figure 3B**). The results revealed 10 conserved motifs. TgMTP5 contained the fewest motifs with only motif 4 among all TgMTP family members. Motif 1 was only present in TgMTP members belonging to the Mn-CDF subfamily and TgMTP6.1, 6.2, 6.3 of the Fe/Zn-CDF subfamily. Motif 2 existed in all TgMTPs of the Fe/Zn-CDF and Zn-CDF subfamilies but not in the Mn-CDF subfamily. Motif 3 was specific to the Fe/Zn-CDF subfamily members. Motif 7 was only found in TgMTP6.1, 6.2 and 6.3 of the Fe/Zn-CDF subfamily. Motif 4 and 6 were absent only in TgMTP6.1, 6.2 and 6.3. Motif 5 was unique to TgMTP7.1, 7.2 and 7.3 of the Fe/Zn-CDF subfamily. Motifs 9 and 10 were present in all TgMTP members except the Mn-CDF subfamily. The sequences of all motifs are shown in **Supplementary Figure 1**.

**Figure 3C** displays two major protein domains (PF16916 and PF01545) in the TgMTP family. PF16916 only existed in the Mn-CDF clade and TgMTP6.1-6.3, while PF01545 distributed across all members.

Gene structure analysis (**Figure 3D**) showed that *TgMTP* members of the same clade had higher consistency in CDS length, such as *TgMTP6.1*, *TgMTP6.2* and *TgMTP6.3*. Moreover, as the identification was based on full-length transcriptome, all members displayed only one CDS region and UTR region, without intron region.

### 3.4 Ka/Ks analysis of TgMTP genes

The Ka/Ks analysis revealed varied selection pressures among *TgMTP* gene pairs (**Table 2**). The Ka/Ks ratio of *TgMTP1* and *TgMTP2* was 0.166849, significantly lower than 1, indicating purifying selection between these two members. *TgMTP6.1* and *TgMTP6.2* had a Ka/Ks ratio of 0.565191, while *TgMTP6.1* and *TgMTP6.3* showed a Ka/Ks ratio of 0.680997, slightly higher than 0.5, suggesting relatively weak positive selection. Notably, an extremely low Ka/Ks value of 0.0700296 was observed between *TgMTP7.1* and *TgMTP7.2*, evidencing robust purifying selection. *TgMTP7.2* and *TgMTP7.3* also exhibited a Ka/Ks ratio of 0.194371, lower than 1, implying purifying selection as well. Collectively, most *TgMTP* gene pairs were under various extents of purifying selection, reflecting functional differentiation and constraints among family members. However, a relaxation of selective pressure was observed between *TgMTP6.1/6.2* and *TgMTP6.1/6.3*. Overall, this analysis provided insights into the molecular evolution patterns and selection forces shaping diversity and conservation within the *TgMTP* gene family.

**Table 2:** Ka/ks value of *TgMTP* genes.

Gene 1	Gene 2	Ka	Ks	Ka/Ks	*P*-Value(Fisher)	Length
*TgMTP1*	*TgMTP2*	0.00212382	0.012729	0.166849	0.0377915	1260
*TgMTP6.1*	*TgMTP6.2*	0.011771	0.0208265	0.565191	0.302987	768
*TgMTP6.1*	*TgMTP6.3*	0.0138441	0.0203291	0.680997	0.516173	768
*TgMTP7.1*	*TgMTP7.2*	0.00123525	0.017639	0.0700296	0.00536092	1098
*TgMTP7.2*	*TgMTP7.3*	0.00212509	0.0109332	0.194371	0.0555731	1311

### 3.5 GO annotation of TgMTP genes

**Figure 4:**
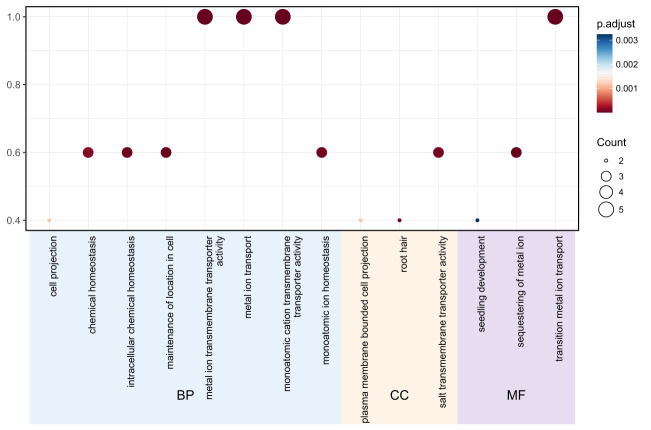
Gene ontology analysis of the *TgMTP* gene family.

Gene ontology (GO) analysis was performed to predict the functional roles of the 11 *TgMTP* members. As shown in **Figure 4**, the significantly enriched GO terms could be categorized into biological process (BP), cellular component (CC) and molecular function (MF). For BP, the major terms included metal ion transport, metal ion homeostasis, transition metal ion transport and sequestering of metal ion. Other notable terms were intracellular chemical homeostasis, chemical homeostasis and seedling development. In the CC category, key enriched terms were plasma membrane bounded cell projection and root hair. The predominant MF terms were metal ion transmembrane transporter activity, monoatomic cation transmembrane transporter activity and monoatomic ion homeostasis. These results indicate that the TgMTP family is associated with metal transport, homeostasis and sequestration, consistent with their predicted roles as metal cation transporters. The GO terms related to root and plasma membrane localization further suggest their functional importance in roots for metal acquisition and compartmentalization. This GO analysis provides an overview of putative biological roles of the TgMTP transporters and will inform further investigations into their metal transport capabilities and physiological functions in tulip.

### 3.6 miRNA regulation network for TgMTP genes

**Figure 5:**
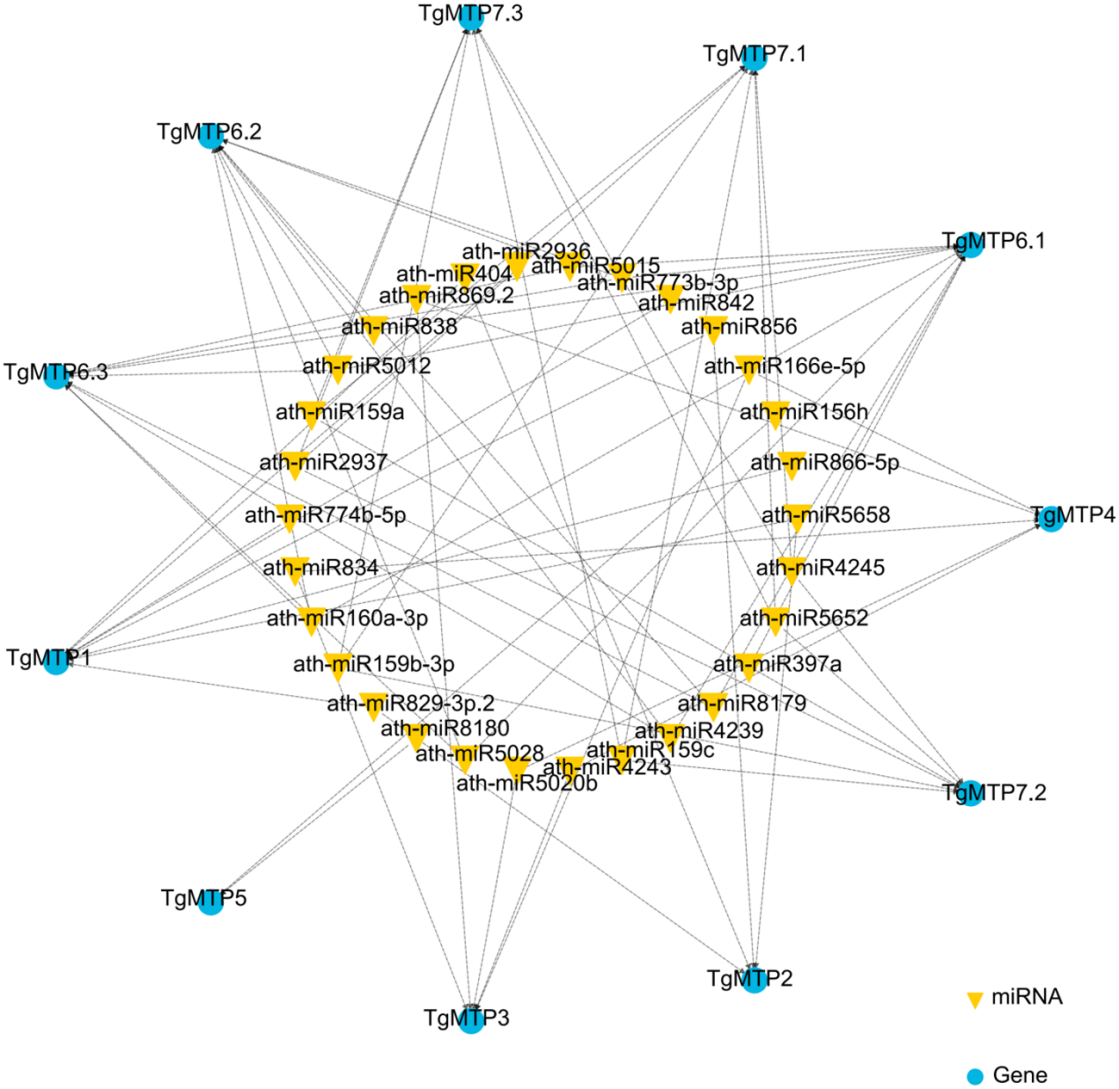
Regulation network of miRNA-*TgMTP* genes.

We predicted the potential miRNA binding sites of *TgMTP*s using psRNATarget (Dai and Zhao, 2011) In total, 30 miRNAs were identified that may regulate *TgMTP*s (**Figure 5**; **Supplementary Table 1**). Most miRNAs had multiple *TgMTP* targets, such as miR159a, which could target 3 *TgMTP* genes (**Figure 5** and **Supplementary Table 1**). In contrast, some *TgMTP*s could be targeted by several miRNAs. For instance, *TgMTP7.1* could be targeted by 6 miRNAs, including ath-miR159a, ath-miR159b-3p and ath-miR159c (**Figure 5**; **Supplementary Table 1**). The relationships between *TgMTP*s and miRNAs warrant further investigation.

### 3.7 qRT-PCR analysis of TgMTP genes under metal treatment

**Figure 6:**
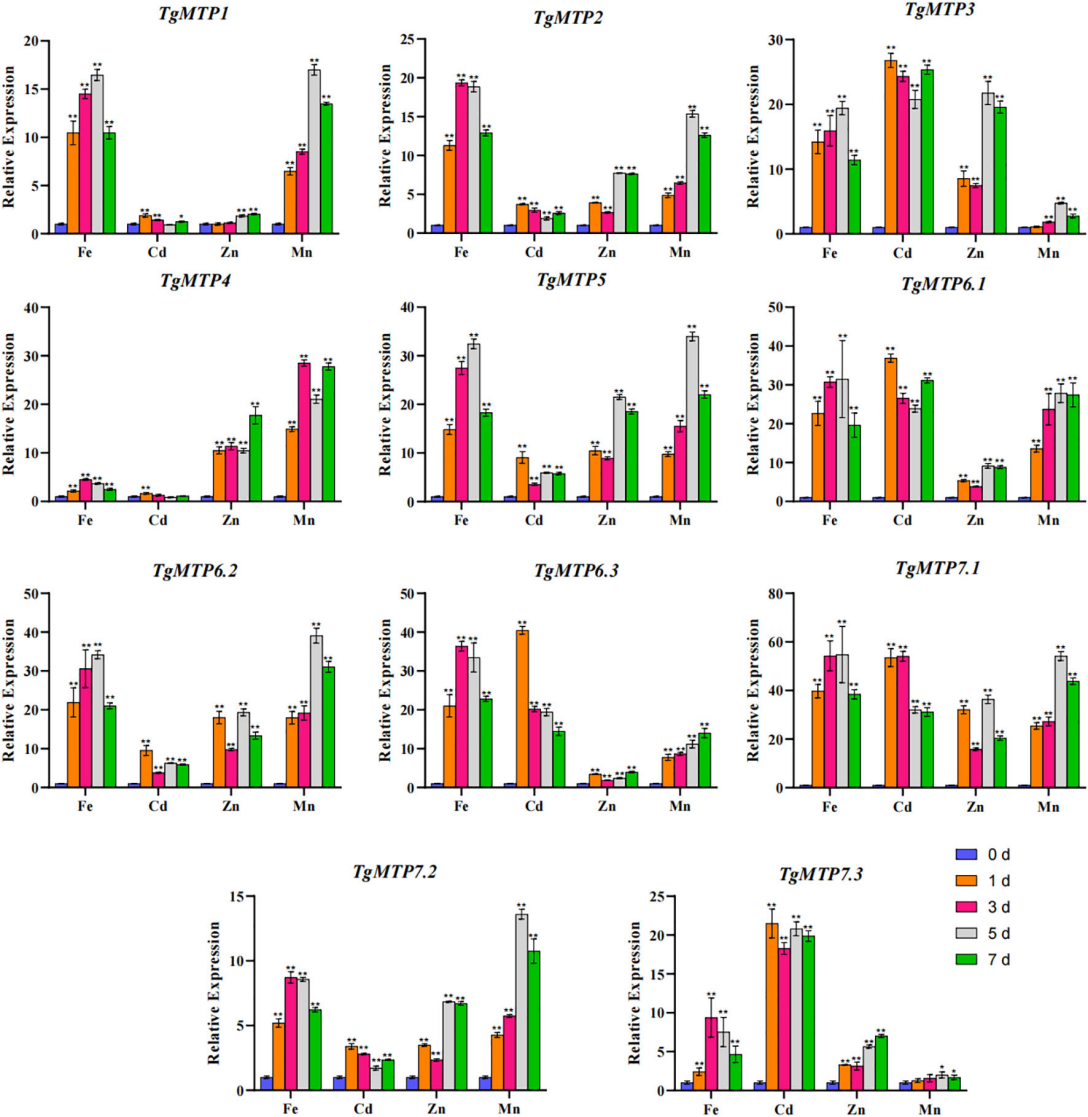
Relative expression level of *TgMTP* genes under different metal treatment. Two-way ANOVA analysis was used to compare the expression level.Data represent means ± SD, n = 3. * means *p*<0.05, ** means *p*<0.001.

The relative expression profiles of 11 *TgMTP* genes in response to Fe^2+^, Mn^2+^, Cd^2+^ and Zn^2+^ treatments are shown in **Figure 6**. The transcript levels were examined by qRT-PCR at 0, 1, 3, 5 and 7 days after metal exposure. Significant variations in expression were observed among *TgMTP* genes by metal treatments over time. Overall, most TgMTPs were markedly induced by four metal (except *TgMTP4* under Cd treatment), especially at later time points for most *TgMTP* genes. For instance, *TgMTP7.1* displayed over 30-fold upregulation after Cd treatment for 1, 3, 5 and 7 days compared to 0 day. Substantial stimulation of transcript levels was also evident for *TgMTP3*, *TgMTP6.1* and *TgMTP6.3* following Cd exposure. For Fe^2+^ treatment, 72.7% (8/11) of the *TgMTP* genes exhibited over 10-fold upregulation in expression. Similarly, greater than 10-fold increased expression was observed for 54.5% (6/11) and 81.8% (9/11) of the genes under Zn^2+^ and Mn^2+^ exposure, respectively. These results demonstrate that most *TgMTP*s are responsive to heavy metal stresses. However, the percentage of *TgMTP* genes that could be markedly induced (with relative expression elevated by over 10-fold) by Cd^2+^ treatment was 45.4% (5/11). The data reveals the potential roles of these *TgMTP* genes in metal tolerance and accumulation in tulip. Further functional analyses of the responsive transporters such as *TgMTP7.1* may provide insights into heavy metal detoxification mechanisms in this species.

### 3.8 Heterologous expression of TgMTP7.1

**Figure 7:**
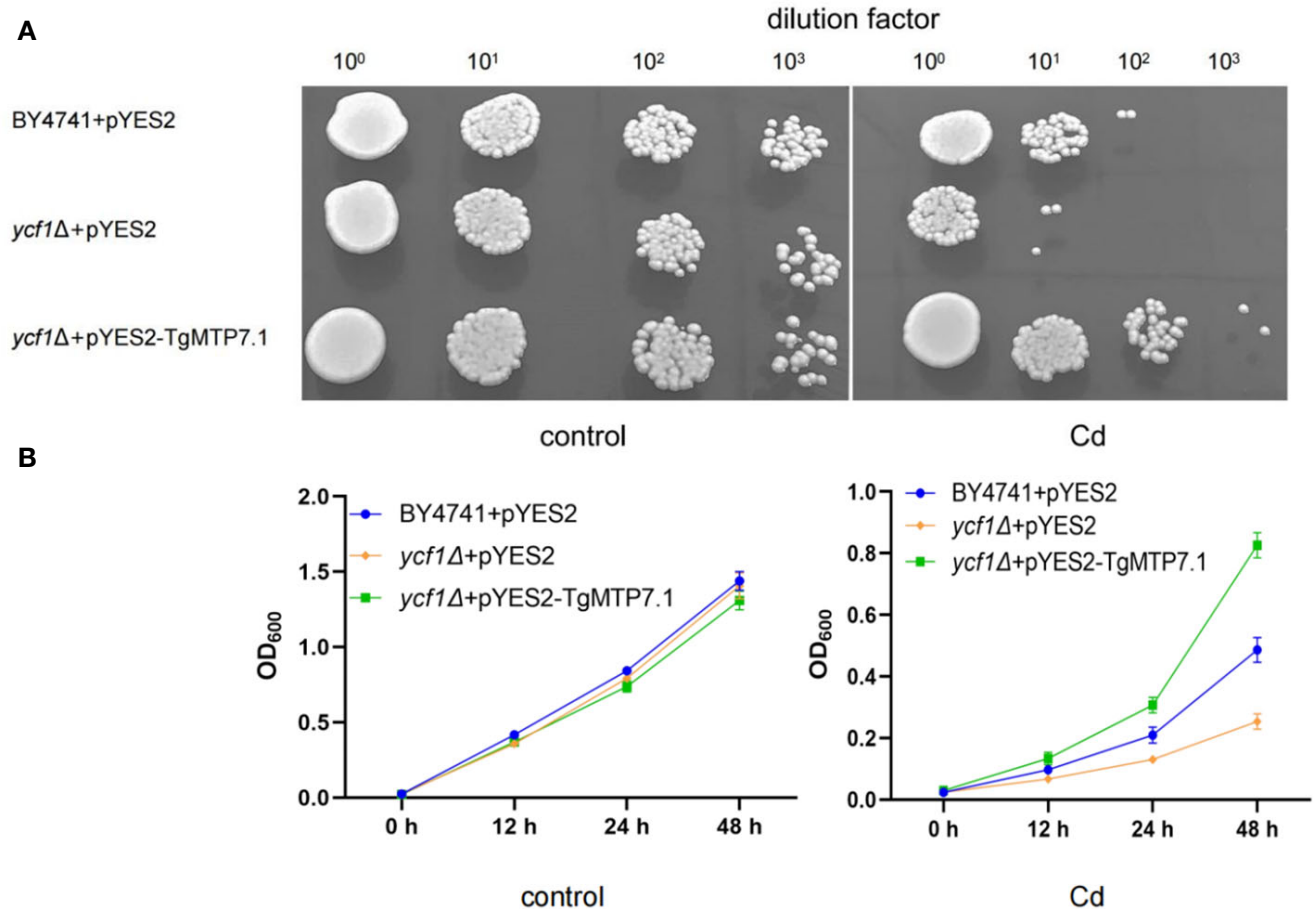
Sensitivity to Cd in yeast. **(A)** Complementation of yeast mutants on solid medium containing heavy metals. Yeast wild-type strain BY4741 was transformed with the empty vector pYES2, Cadmium-sensitive yeast mutants were transformed with either the same empty vector pYES2 or the pYES2 vector harboring the *TgMTP7.1* expression cassette. Transformants were cultured to mid-logarithmic phase at OD_600_ of 0.2 before preparation of 10-fold serial dilutions spanning four orders of magnitude. Subsequently, 2 μL of each diluted sample was pipetted onto medium plates supplemented with 5 mM CdCl_2_ to assess growth phenotypes. **(B)** Sensitivity to Cd when grown in liquid medium contain CdCl2, error bar = mean ± SD, n=3.

The *TgMTP7.1* gene was selected for functional validation as it exhibited the most pronounced upregulation upon Cd treatment when compared with other *TgMTP* genes. The *TgMTP7.1* was heterologous expressed in the parental strain BY4741 and a yeast mutants that are highly sensitive to Cd (*ycf1*Δ), respectively. As shown in **Figure 7**, yeast cells transformed with empty vector pYES2 displayed severely inhibited growth on the medium containing 5 mM CdCl_2_, whether in plates (**Figure 7A**) or liquid medium (**Figure 7B**). In contrast, *ycf1*Δ expressing pYES2-TgMTP7.1 exhibited significantly enhanced tolerance to Cd toxicity. At the 10^2^ dilution factor, the survival rate of the pYES2 transformants (*ycf1*Δ+pYES2) was 0, and at the 10^3^ dilution factor, the survival rate of the pYES2 transformants (BY4741+pYES2) was 0. Whereas yeast cells with TgMTP7.1 (*ycf1*Δ+pYES2-TgMTP7.1) grew readily at dilutions of 10^1^ and 10^2^, even the dilutions of 10^3^, the *ycf1*Δ+pYES2-TgMTP7.1 transformants showed a survival. These results demonstrate that *TgMTP7.1* expression could effectively rescue the Cd sensitivity of yeast cells. The increased Cd tolerance conferred by *TgMTP7.1* heterologous expression indicates it possesses Cd transport activity and likely functions in vacuolar sequestration of Cd in plants.

## 4 Discussion

Mn, Zn, and Fe are trace elements critical for proper growth, development, function, and sustainment of plants. Notwithstanding their significance in many biochemical processes, superfluous quantities of these elements can be toxic to plants, eliciting decreases in agricultural yield and quality (Ghori et al., 2019). Meanwhile, other non-essential elements, including cadmium (Cd) can likewise be absorbed and elicit toxicity even at very low concentrations (Clemens, 2001). Cd exposure elicits toxicity symptoms in plants such as growth inhibition, declines in photosynthesis and pigmentation, cellular damage from oxidative stress, and antagonistic effects on the uptake of essential nutrients (Mishra et al., 2006; Mobin and Khan, 2007; Wang et al., 2009).

Concomitant with the progression of industrialization, heavy metals (particularly Cd) are amassing abundantly in the environment. These elements ingress plants and other organisms through the food chain, eliciting toxicity symptoms even at diminished concentrations. This phenomenon poses a substantial hazard to ecosystems and may also imperil human wellbeing (El-Sappah et al., 2012; Elrys et al., 2018). Ornamental plants, not intended for human consumption, demonstrate a robust potential for the remediation of heavy metal-contaminated soils, given their ability to thrive in such environments (Khan et al., 2021). The *MTP* gene, which functions in the transport of metal ions within plants, plays a crucial role in the resistance of plants to heavy metals (Ricachenevsky et al., 2013). However, research on the *MTP* gene family in tulips, as one of the significant ornamental plants, has not yet been initiated.

In this study, we performed a comprehensive genome-wide identification and characterization of the *MTP* gene family in tulip. A total of 11 *TgMTP* members were identified based on conserved MTP/CDF domains and phylogenetic analysis. The nomenclature and classification of *TgMTP*s were assigned according to sequence homology with *A. thaliana* and rice *MTP*s *(* Mäser et al, 2001). As the first investigation of *MTP*s in tulip, we comprehensively studied the genomic organization, evolutionary relationships, structural features and expression patterns of *TgMTP*.

Phylogenetic analysis clustered the 11 TgMTPs into three distinct subfamilies (**Table 1**), together with MTP orthologs from soybean, tomato, rice and *A. thaliana* (**Figure 1**) (Montanini et al., 2007; Gustin et al., 2011; Migocka, 2015). This suggests that the divergence of plant MTPs into different subfamilies preceded the speciation of monocot and dicot plants. In tomatoes, 11 MTP proteins are identified, six of which may play a significant role in the response to heavy metal stress (El-Sappah et al., 2021). Similarly, this study has also identified five *TgMTP* genes that exhibit a strong response to Cd (**Figure 6**). The phylogenetic classifications of TgMTPs provide a foundation to investigate their functional specificities.

Conserved motif analysis revealed shared motifs between TgMTPs of the same subfamily, implying similar structure-function relationships. Distinct motifs among subfamilies may contribute to functional divergences. The predominant α-helical transmembrane topology is consistent with their predicted roles as membrane transporters (El-Sappah et al., 2021). Differences in secondary structure composition and length of hydrophilic loops connecting the TMs could impact substrate access, transport kinetics and metal selectivity (El-Sappah et al., 2021). The two duplicated pseudosymmetrical domains (TMs 1-3 and TMs 2-4-5-6) form the translocation pathway and mediate alternating access of metals. Further mutational and structural analyses are required to elucidate the structure-function relationships of TgMTPs.

Varied subcellular localizations of TgMTPs were predicted, including vacuolar membrane, plasma membrane and cytoplasm (**Figure 2**). Similar localizations have been confirmed for MTPs in *A. thaliana*, soybean and rice (Delhaize et al., 2003; Yuan et al., 2012; Zhang et al., 2020). The vacuolar TgMTPs likely sequester toxic metals like Cd into vacuoles. Plasma membrane TgMTPs may regulate metal uptake and efflux. Whereas cytoplasmic members could be involved in intracellular metal transfer or storage. Determining the precise localizations and tissue expression patterns of *TgMTP*s will clarify their physiological roles in tulip. The construction of miRNA-gene regulatory networks may also engender avenues for investigating the mechanisms of action of *TgMTP*s, particularly miRNA159 (targets *TgMTP7.1*) which has been implicated in abiotic stress tolerance in sugarcane (Patade and Suprasanna, 2010) and rice (Mohsenifard et al., 2017).

Most *MTP* genes exhibited metal-responsive expression changes, especially induction by Cd (Sasaki et al., 2014). The considerable stimulation of predominant *TgMTP*s like *TgMTP7.1* under Cd implies their importance for metal tolerance and sequestration in tulip (**Figure 6**). Further functional analyses of these metal and responsive transporters will uncover their contribution to low Cd traits in tulip.

Notably, heterologous expression of *TgMTP7.1* could rescue the Cd sensitivity of yeast cells. Enhanced Cd tolerance indicates TgMTP7.1 possesses Cd transport activity in plants, likely for vacuolar sequestration (Delhaize et al., 2003). Characterization of *TgMTP7.1* and other responsive *TgMTP*s will help elucidate the mechanistic basis of heavy metal homeostasis and distribution in tulip. Engineering key *TgMTP*s may generate tulip cultivars with reduced heavy metal accumulation or heavy metal tolerance to address and mitigate soil cadmium contamination.

Tulip is an economically important ornamental geophyte cultivated worldwide, with bulbs prone to accumulating toxic heavy metals that pose potential toxicity risks (Byczyńska et al., 2019). In wheat, by genetic diversity could be exploited for developing low-metal wheat cultivars through molecular breeding approaches (Zaid et al., 2018). Our study represents the first genome-wide analysis of MTP transporters that underlie heavy metal accumulation in tulip. The results lay an important foundation for elucidating the transporters and molecular mechanisms governing metal distribution and detoxification in this species.

Notably, several Cd-responsive *TgMTP*s were identified as high priority candidates for functional analyses. Due to the substantial threat that Cd poses to human health (Fowler, 2009). In furture, overexpression or knockdown/knockout of these key transporters in tulip would help establish their importance for vacuolar sequestration and shoot translocation of Cd. Comparing Cd contents and oxidative damage between transgenic and wild-type tulip plants under Cd exposure could reveal impacts on Cd tolerance and accumulation phenotypes. Furthermore, introducing mutations into specific TgMTPs by genome editing may help determine structural domains or residues critical for substrate recognition and transport kinetics. This will play a crucial role in elucidating the mechanisms by which MTP proteins contribute to Cd tolerance or transport in tulips. Concurrently, it offers significant insights for the cultivation of tulip varieties with superior heavy metal remediation capabilities.

In addition to genetic manipulation in tulip itself, heterologous expression also provides a rapid screening method for identifying *MTP* genes with heavy metal tolerance or transport capabilities. Cd-sensitive yeast could be complemented by key *TgMTP*s to assess their ability to restore Cd tolerance. Expressing *TgMTP*s in *A. thaliana* metal hyperaccumulator or hypertolerance mutants would test whether they can alter natural variation in Cd accumulation or sensitivity. Analyzing fluctuations in root/shoot metal contents and mapping *TgMTP* expression patterns in the heterologous hosts could provide deeper insights into transporter roles in metal distribution between tissues and subcellular compartments (Verbruggen et al., 2009).

In summary, this comprehensive study revealed important molecular and functional characterisics of the *TgMTP* gene family as putative key determinants of heavy metal accumulation in tulip. Our results lay a foundation for further elucidating TgMTP transport capabilities, expression regulation and physiological roles. Genetic manipulation in heterologous expression systems present promising approaches to establish structure-function relations and contributions of specific *TgMTP*s to Cd homeostasis. Key *TgMTP*s identified also present potential targets for engineering or breeding low Cd cultivars to reduce metal toxicity risks and enable sustainable production. Further deciphering *TgMTP*-mediated metal transport and detoxification mechanisms will provide deeper insights into heavy metal accumulation traits in geophytes.

## 5 Conclusions

In this study, leveraging Iso-seq data, 11 MTP genes were identified from tulips and categorized into three subfamilies based on phylogenetic relationships. The MTP proteins within the same subfamily exhibit structural similarities, yet they show differential relative expression in response to metal stress. The identification of the key Cd-responsive gene *TgMTP7.1*, along with its heterologous expression in yeast, preliminarily elucidates the function of *TgMTP7.1*. This provides vital genetic material for in-depth future research into the functionality of *MTP* genes. Moreover, this study offers valuable insights into using ornamental plants, specifically tulips, for the phytoremediation of heavy metal-contaminated soils.

## Data availability statement

The original contributions presented in the study are included in the article/**Supplementary Material**. Further inquiries can be directed to the corresponding author.

## Author contributions

JL: Data curation, Writing – original draft. GX: Software, Writing – original draft. YZ: Data curation, Writing – original draft. HZ: Software, Writing – original draft. TW: Data curation, Writing – original draft. ZT: Conceptualization, Writing – review & editing. LQ: Conceptualization, Writing – original draft, Writing – review & editing.
