# Supplementary material for: Genome-wide identification, expression and function analysis of the MTP gene family in tulip (Tulipa gesneriana)
Source: Front Plant Sci. 2024 Feb 19;15:1346255. doi: 10.3389/fpls.2024.1346255 (PMC10910078; doi:10.3389/fpls.2024.1346255)
Supplement: Supplementary file 1 [file DataSheet_1.docx]

| 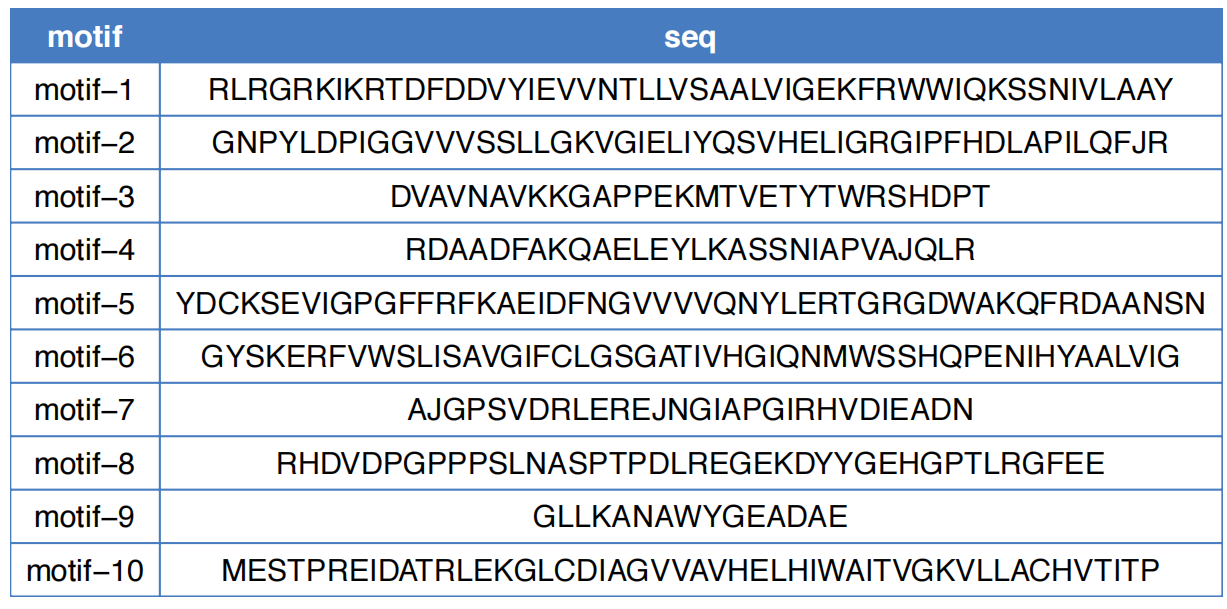 |
| --- |
| Fig. S1 Motif sequence of ten motifs in TgMTP proteins. |

| 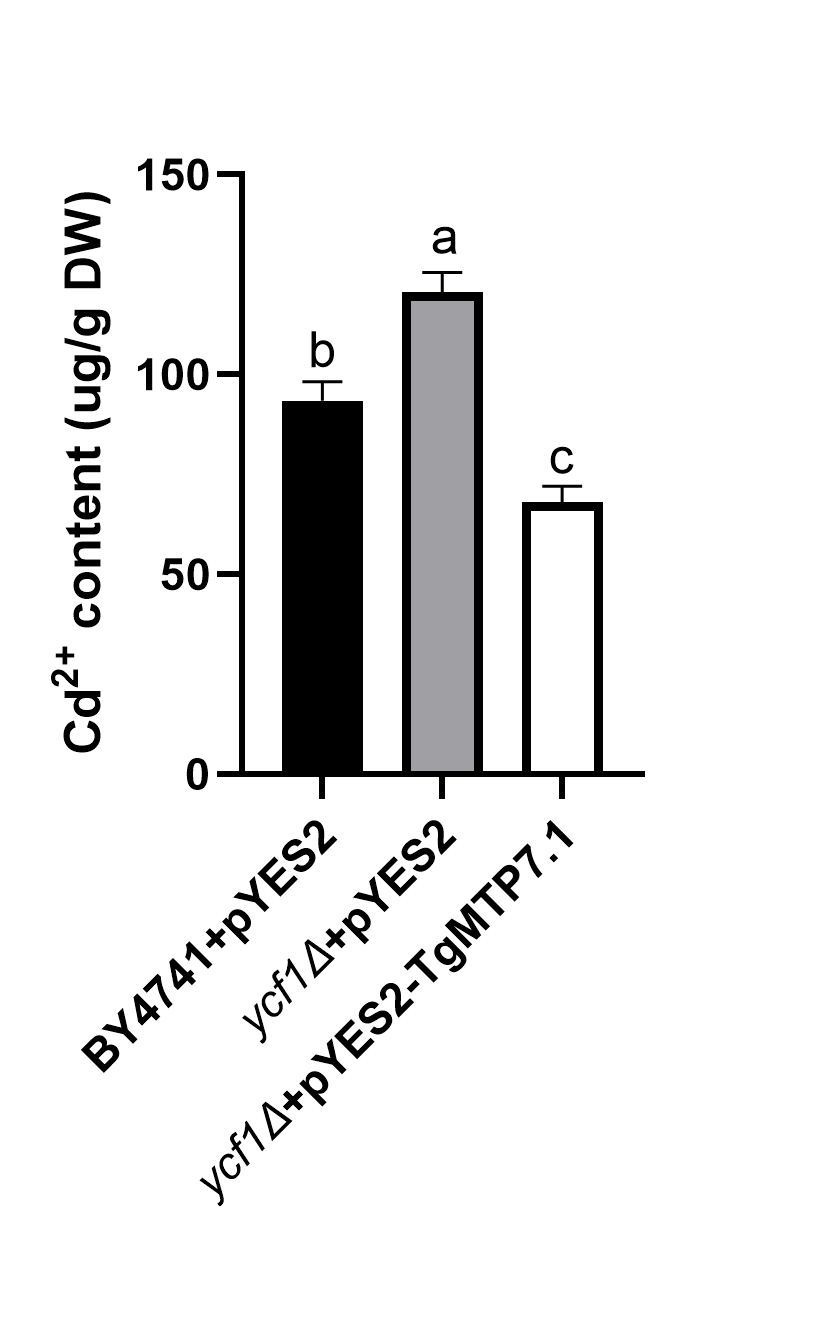 |
| --- |
| Fig. S2 Cd accumulation in the yeast strain BY4741+pYES2, *ycf1Δ*+pYES2, and *ycf1Δ*+pYES2-TgMTP7.1. Bars indicate the standard error of the mean (n = 6). Significant differences between three groups are indicated by different lowercase letters (*P* < 0.01). |

| 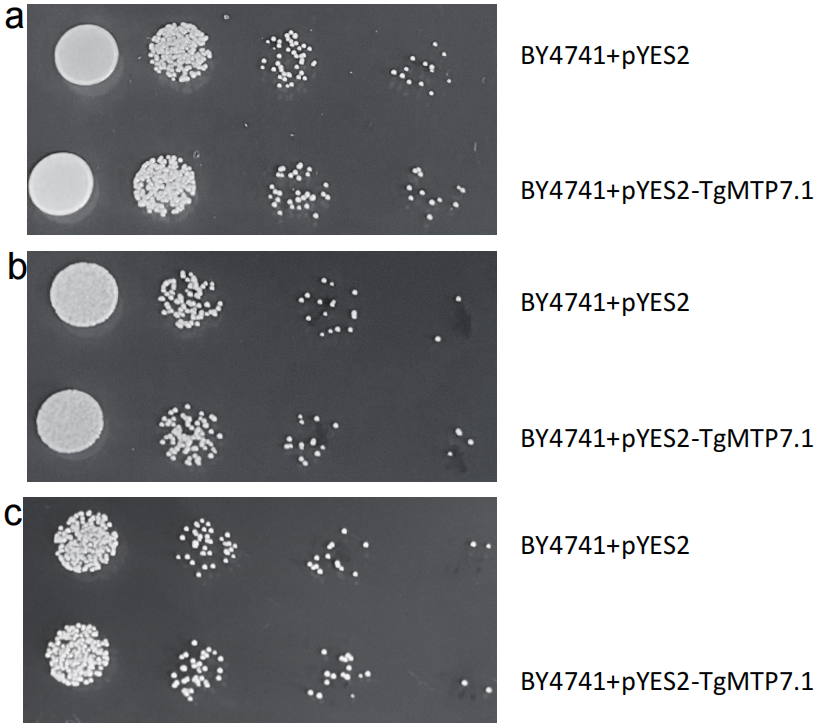 |
| --- |
| Fig. S3 The consequence of TgMTP7.1 expression on Mn, Fe, and Zn endurance in yeast. Transformants were cultured to mid-logarithmic phase at OD_600_ of 0.2 before preparation of 10-fold serial dilutions spanning four orders of magnitude. Subsequently, 2 μL of each diluted sample was pipetted onto medium plates supplemented with 10 mM MnSO_4_ (a), 10 mM FeSO_4_ (b) and 10 mM ZnSO_4_ (c) to assess growth phenotypes. |
